# Investigation of the genetic variation in ACE2 on the structural recognition by the novel coronavirus (SARS-CoV-2)

**DOI:** 10.1186/s12967-020-02486-7

**Published:** 2020-08-24

**Authors:** Xingyi Guo, Zhishan Chen, Yumin Xia, Weiqiang Lin, Hongzhi Li

**Affiliations:** 1 Division of Epidemiology, Department of Medicine, Vanderbilt Epidemiology Center, Vanderbilt University School of Medicine, Nashville, TN 37203 USA; 2 Department of Biomedical Informatics and Vanderbilt-Ingram Cancer Center, Vanderbilt University School of Medicine, Nashville, TN 37203 USA; 3 Department of Dermatology, The Second Affiliated Hospital of Xi’an Jiaotong University, 157 Xiwu Road, Xi’an, 710004 China; 4 The First Affiliated Hospital, Institute of Translational Medicine, Zhejiang University School of Medicine, Hangzhou, 310029 China; 5 Department of Molecular Medicine, City of Hope National Medical Center, Duarte, CA 91008 USA

**Keywords:** COVID-19, ACE2, SARS-CoV-2, S-protein, Missense

## Abstract

**Background:**

The outbreak of coronavirus disease (COVID-19) was caused by severe acute respiratory syndrome coronavirus 2 (SARS-CoV-2), through its surface spike glycoprotein (S-protein) recognition on the receptor Angiotensin-converting enzyme 2 (ACE2) in humans. However, it remains unclear how genetic variations in ACE2 may affect its function and structure, and consequently alter the recognition by SARS-CoV-2.

**Methods:**

We have systemically characterized missense variants in the gene ACE2 using data from the Genome Aggregation Database (gnomAD; N = 141,456). To investigate the putative deleterious role of missense variants, six existing functional prediction tools were applied to evaluate their impact. We further analyzed the structural flexibility of ACE2 and its protein–protein interface with the S-protein of SARS-CoV-2 using our developed Legion Interfaces Analysis (LiAn) program.

**Results:**

Here, we characterized a total of 12 ACE2 putative deleterious missense variants. Of those 12 variants, we further showed that p.His378Arg could directly weaken the binding of catalytic metal atom to decrease ACE2 activity and p.Ser19Pro could distort the most important helix to the S-protein. Another seven missense variants may affect secondary structures (i.e. p.Gly211Arg; p.Asp206Gly; p.Arg219Cys; p.Arg219His, p.Lys341Arg, p.Ile468Val, and p.Ser547Cys), whereas p.Ile468Val with AF = 0.01 is only present in Asian.

**Conclusions:**

We provide strong evidence of putative deleterious missense variants in ACE2 that are present in specific populations, which could disrupt the function and structure of ACE2. These findings provide novel insight into the genetic variation in ACE2 which may affect the SARS-CoV-2 recognition and infection, and COVID-19 susceptibility and treatment.

## Background

The outbreak of the coronavirus disease 2019 (COVID-19), caused by a novel (new) coronavirus (SARS-CoV-2), has been characterized as a global pandemic [1–5]. COVID-19 is rapidly spreading across the world and affecting all populations. It has been documented that the S-protein of SARS-CoV-2 plays a key role in the recognition to the peptidase domain (PD) of the Angiotensin converting enzyme **(**ACE2) in humans [6, 7]. The three-dimensional protein structures of SARS-CoV-2 have recently been determined, which provide important insight into the treatment of the disease, such as vaccine development, antibody design and drug discovery [7]. The first X-ray crystallization structure of 3CLpro was resolved by Liu et al. at 2.16 Å resolution (Protein data bank (PDB) id 6lu7). The virus S-protein structure was first observed by Wrapp et al. at 3.46 Å resolution by electron microscopy (PDB id 6vsb) [8]. The first full-length S-protein in complex with human ACE2 Cryo-EM structure was observed by the institute of Xihu University [7], and at almost the same time, the X-ray structure of the S-protein RBD domain in complex with ACE2 was solved by Tsinghua University at 2.45 Å re solution [9]. Two X-ray structures of S2 subunit have been determined by Zhu et al. (PDB id 6lxt and 6lvn). In addition to SARS-CoV-2, several three-dimensional structures of ACE2, especially in complex with SARS S-protein, have been solved. It shows that ACE2 structure is flexible to toggle between open and close states when it binds an inhibitor or virus S-protein. Genetic variation, especially deleterious missense variants in these flexible regions, may affect its function and structure, and consequently alter the recognition by SARS-CoV-2. Thus, it’s important to systematically characterize and evaluate potentially deleterious variants in ACE2, which may affect SARS-CoV-2 recognition and infection, and COVID-19 susceptibility and treatment.

## Methods

### Characterization of genetic variants in ACE2 from the genome Aggregation Database

The genome Aggregation Database (gnomAD v2.1.1) has provided summary data (i.e. allele counts) for germline variants from 125,748 WES and 15,708 whole-genome sequences from unrelated individuals, sequenced as part of various disease-specific and population genetic studies, through the website browser http://gnomad.broadinstitute.org/. We characterized a total of 251 non-silent genetic variants, including missense senses, splicing, stop gain/loss and frameshift/inframe deletion, located in ACE2 genes, after removing those with low quality control or had a allele frequency (AF) = 0 (Additional file 1). Next, we focused on just the top 21 ACE2 missense variants with an AF > 8 × 10^−5^ in combining populations, which account for the major proportion of subjects carrying non-silent variants (Additional file 1).

### Variant annotation, bioinformatics and statistical analyses

The ANNOVAR tool [10] was applied to annotate missense and disruptive variants. Disruptive variants were defined by nonsense, splice-site and frameshift. To further evaluate the functional impact of missense variants, we annotated each variant with the possible impact of an amino acid substitution on the structure/function from five protein prediction algorithms, including Polyphen-2 HumDiv, Poplyphen HumVar, Sorting Intolerant From Tolerant (SIFT), logistic regression test scores and MutationTaster. Of the top 21 ACE2 missense variants with a AF > 8 × 10^−5^ in combining populations, only putative deleterious missense variants predicted by at least two tools were further analyzed.

### Protein structure analysis for ACE2 and the interaction between COVID-19 spike glycoprotein and ACE2

The protein structures are downloaded from the RCSB Protein Data Bank or from the authors’ website. The protein structure figures and animations are produced by PyMol and its Morph function [11]. The 2-dimensional interaction diagrams are produced by Schrödinger Maestro software [12]. The 3-dimensional interaction plots are generated by our in-house developed Legion Interfaces Analysis (LiAn) program, which can calculate and display protein–ligand or protein–protein interactions (such as hydrogen bond, salt-bridge, water-bridge, π-interactions, hydrophobic interactions, halogen bond, etc.) for single protein structure or massive structures from molecular dynamics simulations. The LiAn program also integrates protein–protein interface analysis, protein structural clustering, protein interaction energy calculations, and fixed water predictions to analyze large amount of protein structures automatically.

## Results

**Fig. 1:**
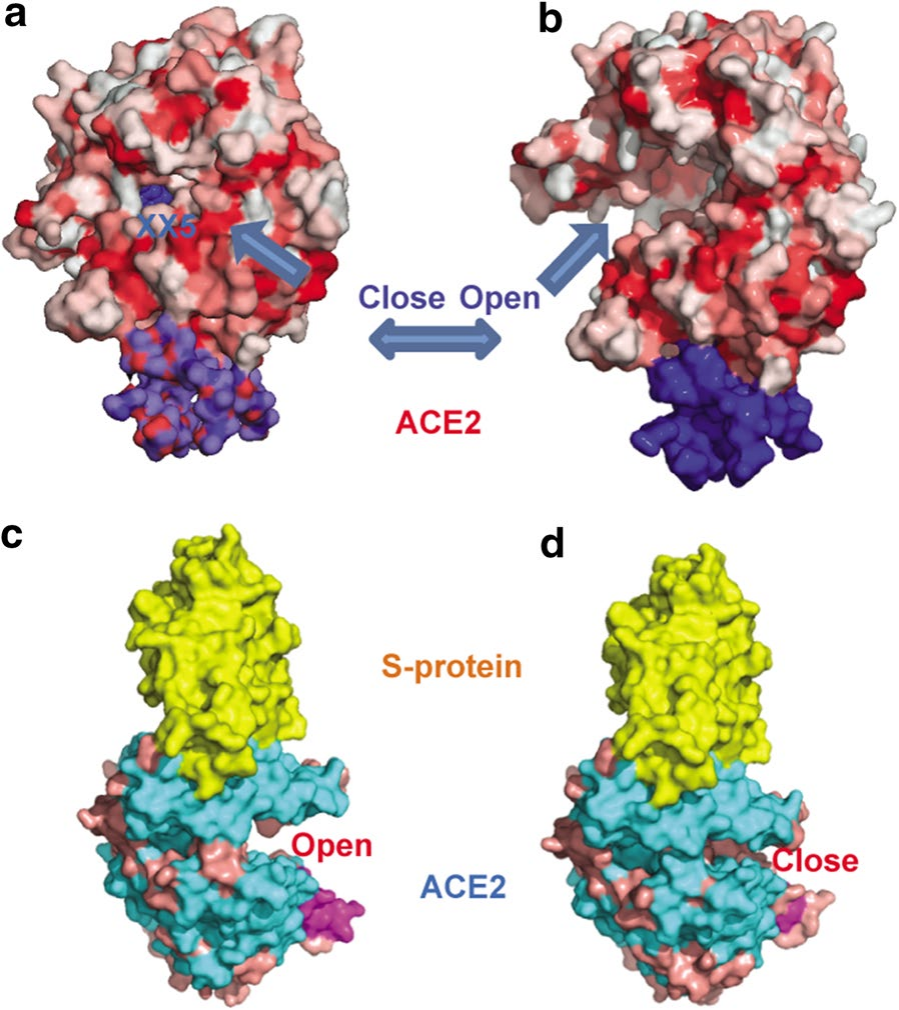
Open/closed state of ACE2 and S-protein binding. **a** Closed state when ACE2 binds MLN-4760 (XX5) inhibitor (PDB1r4l). **b** Open/native state of ACE2 (PDB 1r42). **c** Most PDB structures show that SARS S-protein (colored yellow) binds the open state of ACE2, as from one monomer of PDB 3scl. **d** S-protein shows binding to closed state of ACE2 from another monomer of PDB 3scl

To illustrate how genetic variation may affect the structure, we analyzed the structural interactions between ACE2 and SARS-Cov-2. As displayed in Fig. 1a, b and Additional file 2: Figure S1, we demonstrated that ACE2 has two states, i.e. open and closed, for its native and ligand-binding states through a large hinge-bending motion [13]. In open state, ACE2 opens wide from its active site to wait for a ligand to enter. When the ligand enters ACE2 active site, it triggers ACE2 to close the active slot. Most SARS binding structures (e.g. PDB ids 2ajf, 3d0g, 3kbh, 3scl) show that S-protein binds the open/native state of ACE2. However, as depicted in Fig. 1c, d and Additional file 2: Figure S2, the two monomers in PDB 3scl display that SARS spike proteins can bind either in an open state or in a closed state of ACE2, which implies that the conformational change of ACE2 can be triggered either by an inhibitor from an inner active site or S-protein from an outer PPI (protein–protein interface) site. The huge conformational change of the two states can be up to 14 Å distance shift between Lys341 and Thr129. The two N-terminal helices (Ser19–Asn53, Ile54–Met82) that contact SARS-Cov-2 S-proteins are among the most flexible regions. The hinge movement of the helices pivots on the loop region of Trp83–Asn90. We also observed that the protein–protein interface of the SARS-CoV-2 spike glycoprotein to ACE2 has more hydrophilic residues than hydrophobic ones. The residues in ACE2 within distance of 3 Å to S-protein are Gln24, His34, Asp38, Tyr41, Gln42, Tyr83 and Lys353 to Gly446, Tyr449, Tyr453, Asn487, Thr500 and Gly502. The PPI interface binds with six hydrogen bonds (Gln24–Asn487, Gln42–Gly446, Gln42–Gln498, Lys353–Gly502), a network of π–cation interactions (Tyr41–Gln498, Tyr41–Asn501, Gln42–Tyr449, Tyr83–Asn487, Gln493–His34), one π-stacking interaction (Tyr83–Phe486), and only one hydrophobic interaction pair (Met82–Phe486). In summary, the structural flexibility of ACE2 implies that its structure could be distorted by potentially deleterious missense variants with the altered amino acids in ACE2, which may consequently affect its binding efficiency to the S-protein in the virus.

**Fig. 2:**
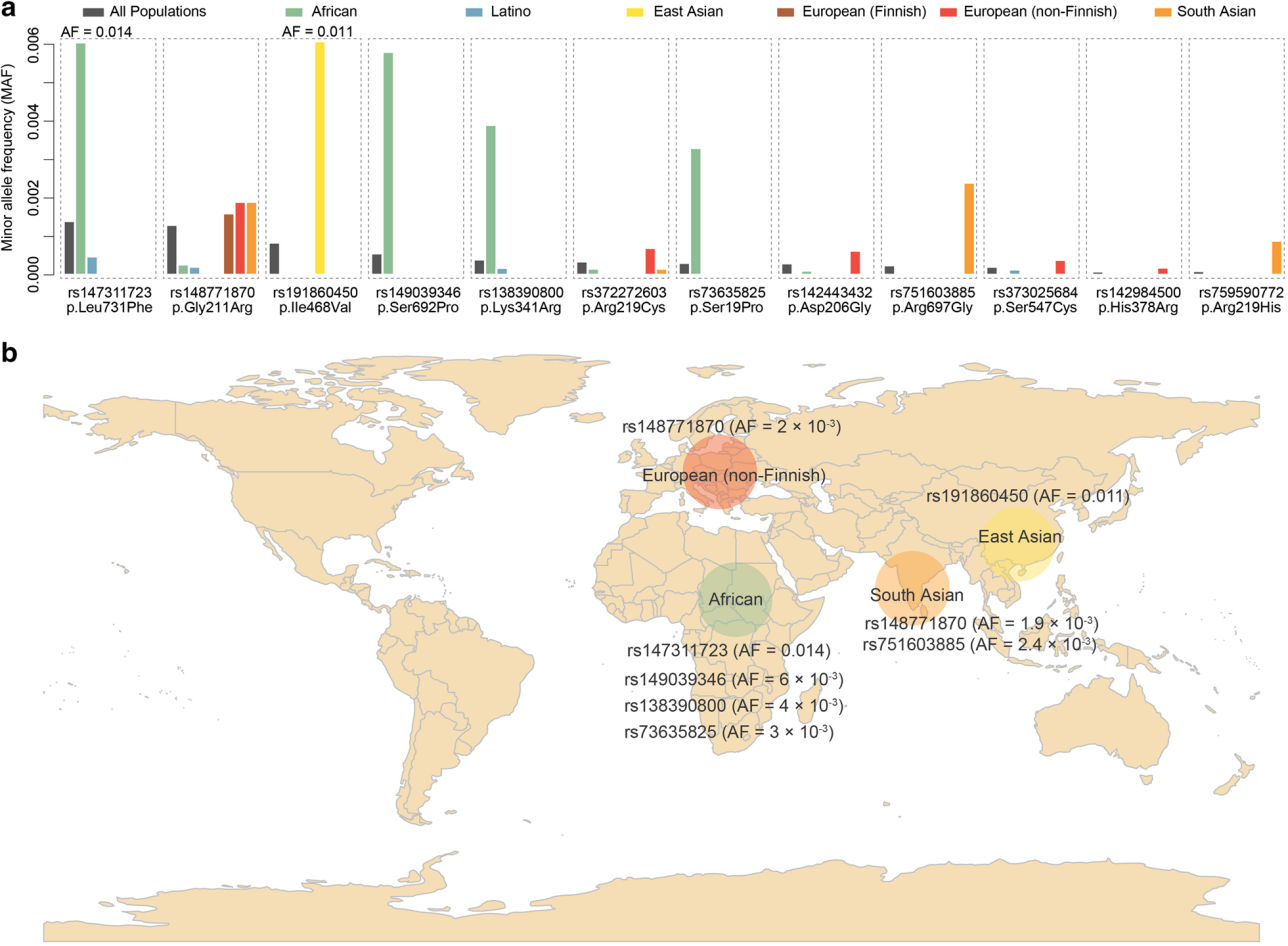
Distribution of the characterized 12 ACE2 missense variants in different populations. **a** AF for each missense variant in different populations. **b** The top AF of the missense variants present in African (rs73635825, rs138390800, rs149039346 and rs147311723), East Asian (rs191860450), South Asian (rs148771870 and rs751603885) and European (rs148771870) populations

We next analyzed germline coding variants in ACE2 from the gnomAD and performed functional predications using six existing bioinformatics tools (see “Methods”). We characterized a total of 12 ACE2 putative deleterious missense variants, whereas the top variants with functional disruptions predicted by all tools included p.Leu731Phe (rs147311723, AF = 0.01 in African), p.Arg219Cys (rs372272603, AF = 7 × 10^−4^ in European), p.Ser547Cys (rs373025684, AF = 4 × 10^−4^ in European) and p.His378Arg (rs142984500, AF = 2 × 10^−4^ in European) (Table 1). Of those, we observed that these variants showed low frequency or rare in all populations (Fig. 2a; Table 1). AF of the characterized variants varied in populations, whereas a majority of them showed population specificities (Fig. 2a). In particular, we observed that two variants with low frequency were present in African (rs147311723) and in East Asian (s191860450), respectively (Fig. 2b). The top AF of the other missense variants were present including, in African (rs73635825, rs138390800, and rs149039346), East Asian (rs191860450), South Asian (rs148771870 and rs751603885) and Europeans (rs148771870) (Fig. 2b).

**Table 1:** Characterization of putative deleterious missense variants in ACE2

rsID	Position (hg19)	Ref	Alt	AA change^a^	Enriched^h^	AF							D^f^	S^g^
						Combined	African	Latino	Asian^b^	European^c^	European^d^	Asian^e^		
**rs147311723**	15582265	G	A	**Leu731Phe**	N/A	1.4 × 10^−3^	0.014	4.8 × 10^−4^	0	0	3.3 × 10^−5^	0	6	N
rs148771870	15607532	C	T	Gly211Arg	N/A	1.3 × 10^−3^	2.7 × 10^−4^	2.1 × 10^−4^	0	1.6 × 10^−3^	1.9 × 10^−3^	1.9 × 10^−3^	2	Y
rs191860450	15593829	T	C	Ile468Val	N/A	8.4 × 10^−4^	0	0	0.011	0	2.2 × 10^−5^	5.6 × 10^−5^	4	Y
rs149039346	15584416	A	G	Ser692Pro	N/A	5.6 × 10^−4^	5.8 × 10^−3^	3.6 × 10^−5^	0	0	2.2 × 10^−5^	5.3 × 10^−5^	4	N
rs138390800	15599392	T	C	Lys341Arg	N/A	4.0 × 10^−4^	3.9 × 10^−3^	1.8 × 10^−4^	0	0	0	0	2	Y
**rs372272603**	15607508	G	A	**Arg219Cys**	N/A	3.5 × 10^−4^	1.6 × 10^−4^	0	0	0	7.0 × 10^−4^	1.6 × 10^−4^	6	Y
rs73635825	15618980	A	G	Ser19Pro	Y	3.1 × 10^−4^	3.3 × 10^−3^	0	0	0	0	0	3	Y^h^
rs142443432	15607546	T	C	Asp206Gly	N/A	3.0 × 10^−4^	1.1 × 10^−4^	3.6 × 10^−5^	0	0	6.3 × 10^−4^	0	2	Y
rs751603885	15584401	T	C	Arg697Gly	N/A	2.5 × 10^−4^	0	0	0	0	0	2.4 × 10^−3^	4	N
**rs373025684**	15590348	G	C	**Ser547Cys**	N/A	2.1 × 10^−4^	0	1.4 × 10^−4^	0	5.4 × 10^−5^	3.9 × 10^−4^	0	6	Y
**rs142984500**	15596376	T	C	**His378Arg**	Y	8.8 × 10^−5^	0	0	0	0	1.9 × 10^−4^	0	6	Y^h^
rs759590772	15607507	C	T	Arg219His	NA	9.8 × 10^−5^	0	0	0	0	1.2 × 10^−5^	8.9 × 10^−4^	5	Y

“a”: AA refers to amino acid

“b”: East Asian

“c”: Finnish

“d”: European not including Finnish

“e”: South Asian

“f”: Number of tools predicted the variant to be deleterious; the top four variants with predicted functional disruptions highlighted in bold

“g”: “Y” refers to The variant likely affecting the protein structure of ACE2, otherwise “N”

“h”: Deep mutagenesis experimental data to show Ser19Pro and His378Arg interactions with SARS-Cov-2 virus from the previous literature [16]

**Fig. 3:**
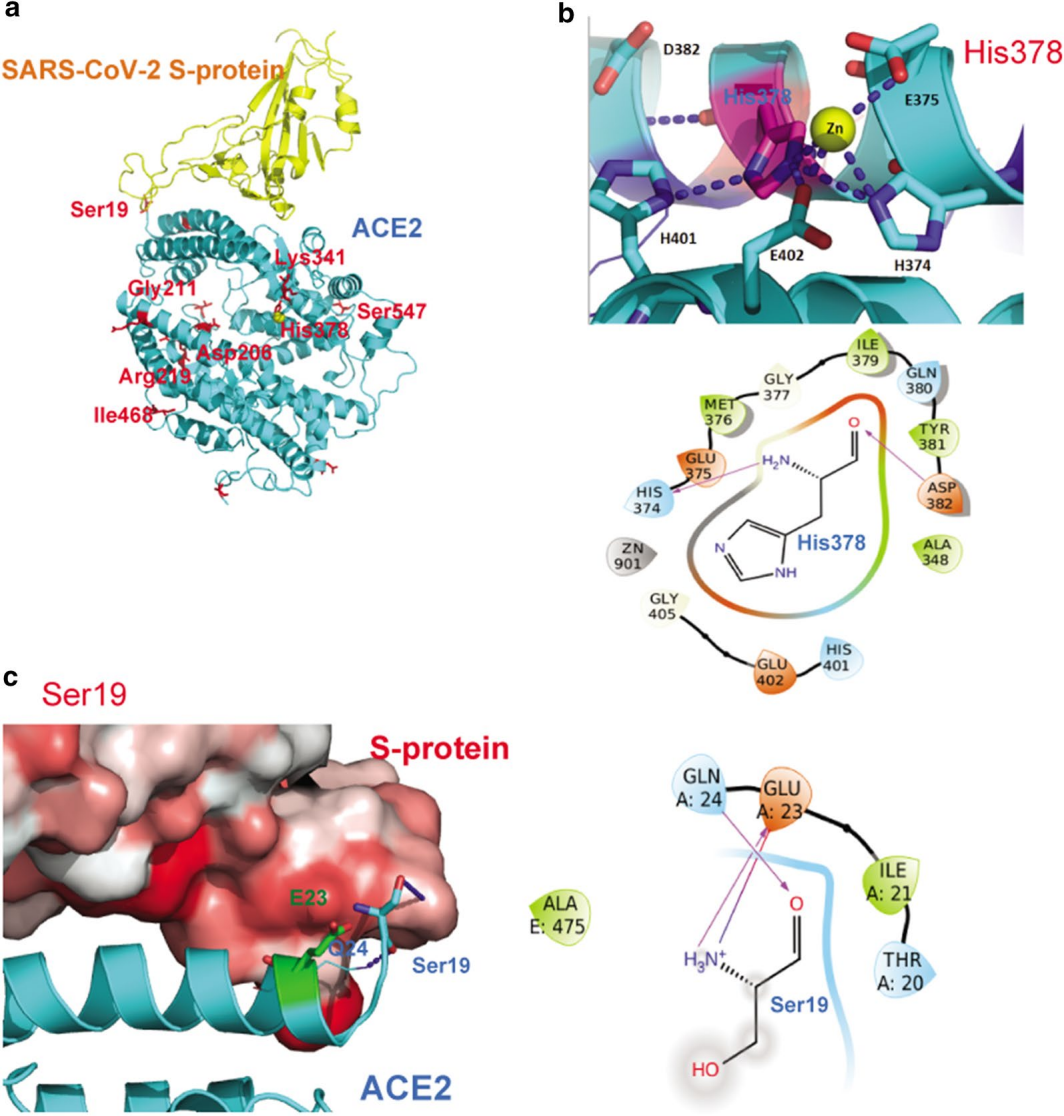
Interaction diagrams for His378Arg (rs142984500) and Ser19Pro (rs73635825). **a** Structural positions of the ACE2 altered amino acids from the nine ACE2 missense variants (colored red). The SARS-Cov-2 S-protein is colored yellow. **b** Interactions of His378Arg and **c** interactions of Ser19Pro. Hydrogen bonds are depicted as blue dots. Π-interactions are depicted as yellow arrows. Hydrophobic interactions are shown as grey arrows. Water molecules from water-bridge are displayed as red spheres

We further analyzed the structural flexibility of ACE2 and its interaction with the RBD of S-protein of SARS-CoV-2 using the two or three-dimensional interaction diagrams (see “Methods”) for nine missense variants on eight residues, as displayed in Fig. 3a. We showed that p.His378Arg could directly weaken the binding of catalytic metal atoms to decrease ACE2 catalytic activity, and p.Ser19Pro (rs73635825, AF = 3 × 10^−3^ in African) could distort the most important helix to interact with the S-protein.

### His378Arg

As shown in Fig. 3b, His378 is a key residue to fix the catalytic metal atom together with Glu375 and Glu402. Its mutation to longer arginine will break the chelation network to Zn atoms, which could result in weakening its peptidase activity. Meanwhile, His378 also stabilizes the structure of the catalytic center via hydrogen bond and π-interaction with Glu402 and His401. Thus, the His378Arg mutant could reduce ACE2 peptidase function and destabilize the ACE2 structure.

### Ser19Pro

Ser19 is the first N-terminal residue that can be shown in an X-ray structure, as displayed in Fig. 3c [9]. It locates at the beginning of helix Ser19-Ile54, which is one of the most important regions to contact virus S-protein. Its backbone forms hydrogen bonds with Glu23 and Gln24 to stabilize the helical structure. It may also interact with the Ser477 in SARS-CoV-2 S-protein through weak hydrophilic interaction. Proline has poor helix-forming propensities, as it either breaks or kinks the helix [14]. Therefore, Ser19Pro mutation could destabilize the helix structure.

We also showed another seven missense variants that may affect secondary structures (i.e. p.Gly211Arg/rs148771870; p.Asp206Gly/rs142443432; p.Arg219Cys/rs759590772; p.Arg219His/rs759590772, p.Lys341Arg/rs138390800, p.Ile468Val/rs191860450, and p.Ser547Cys/rs373025684), whereas p.Ile468Val/rs191860450 with AF = 0.01 is only present in Asian.

### Gly211Arg

**Fig. 4:**
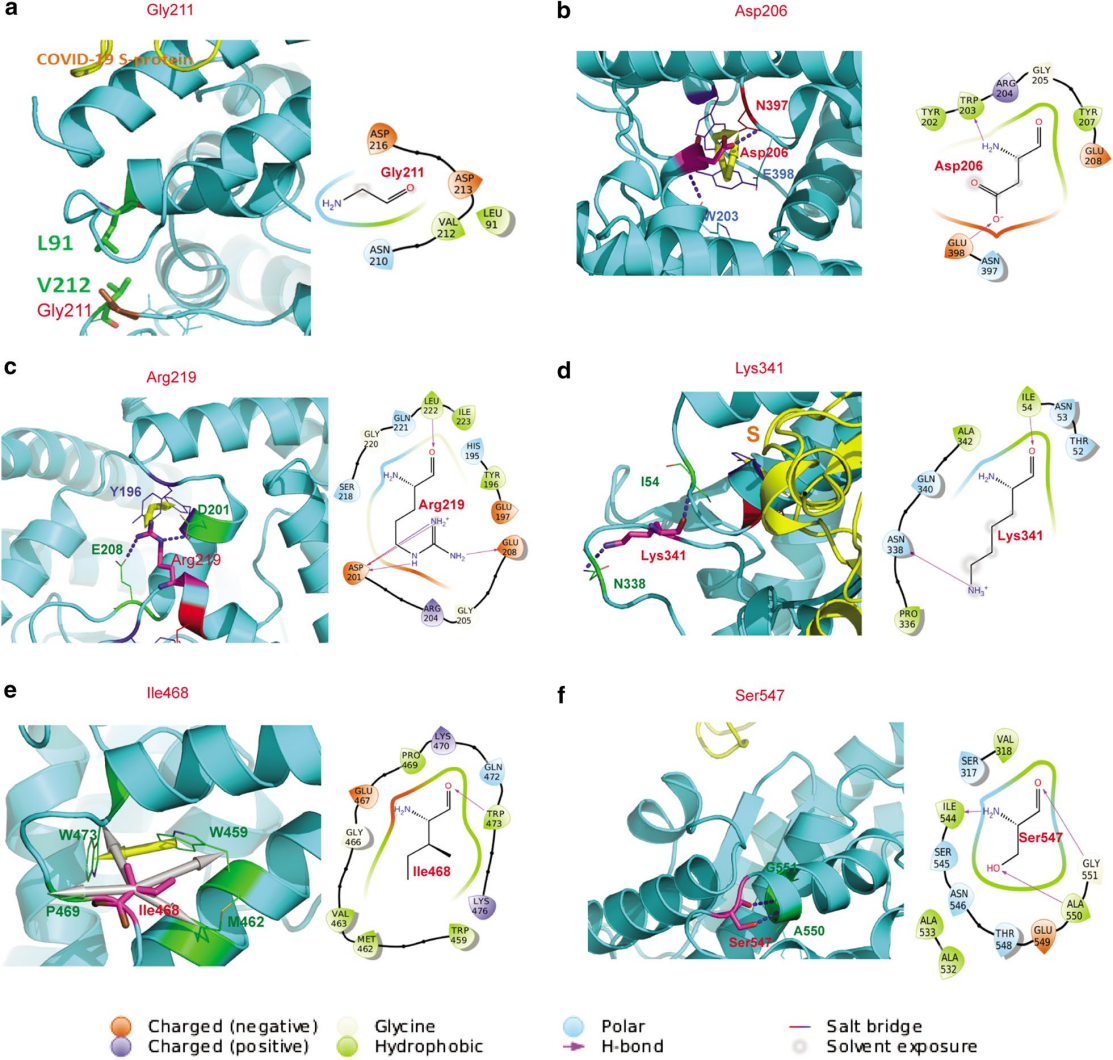
Interaction diagrams for the six residuals from seven *ACE2* missense variants. **a** Interactions of Gly211Arg, **b** interactions of Asp206Gly, **c** interactions of Arg219Cys/His, **d** interactions of Lys341Arg, **e** interactions of Ile468Val and **f** interactions of Ser547Cys. Hydrogen bonds are depicted as blue dots. Π-interactions are depicted as yellow arrows. Hydrophobic interactions are shown as grey arrows. Water molecules from water-bridge are displayed as red spheres

Gly211 is at the turn point of a loop, as depicted in Fig. 4a. Its neighboring Val212 has strong hydrophobic interaction with Leu91 to stabilize the ACE2 structures across secondary structures. Its mutation to long and positive arginine is not favorable for the loop turning. Moreover, its arginine mutation also introduces hydrophilic group to this region, which may weaken the important hydrophobic interaction pair of Val212-Leu91. Therefore, Gly211Arg mutation may destabilize the ACE2 structure.

### Asp206Gly

Asp206 is on a helix of Tyr199–Tyr207 to stabilize multiple secondary structures via a hydrogen bond to Asn397/Glu398, as depicted in Fig. 4b. Its mutation to glycine may affect the ACE2 inhibitor binding site allosterically, as it may disturb the location of the helix of Glu398–Ala413, which is essential for the binding of the catalytic zinc atom.

### Arg219Cys/His

Arg219 is a key center residue to stabilize three helices, i.e. Asp157–Tyr196, Asp198–Glu208, and Arg219–Tyr252. As displayed in Fig. 4c, Arg219 has a strong salt-bridge, hydrogen bond and charge interaction with Asp201 and Glu208, together with cation–π interaction, with Tyr196 across secondary structures to stabilize protein. Its mutation to cysteine or histidine will interrupt the strong interactions and destabilize the protein structure.

### Lys341Arg

Lys341 is on a loop to stabilize another loop from the backbone hydrogen bond to Ile54, as shown in Fig. 4d. It stabilizes the loop structure via a strong hydrogen bond to Asn338. Its mutation to longer arginine may weaken this hydrogen bond and slightly destabilize the loop structure.

### Ile468Val

As demonstrated in Fig. 4e, Ile468 locates at the turn point of a loop to stabilize two helical structures (helices Asp431–Lys465 and Trp473–Val485) via hydrophobic interactions to Trp459, Met462, Pro469 and Trp473, together with a hydrogen bond of its backbone to Trp473. The π-stacking interaction from the pair of Trp459 and Trp473 is an important interaction to bundle the two helices. Ile468 chaperones the pair interaction by fixing the positions of two tryptophans. Consequently, its mutation to valine, which is shorter in side-chain and weaker in hydrophobic interaction, may slightly weaken the contact of two helices and destabilize the protein structure.

### Ser547Cys

As displayed in Fig. 4f, Ser547 stabilizes local helix Ser547–Gly561 through hydrogen bonds to Ala550 and Gly551. Its mutation to cysteine may weaken the hydrogen bond to Ala550 from hydroxyl side-chain to the thiol group, which in turn destabilizes the helical structure slightly.

## Discussion

As shown in Fig. 3, ACE2 has flexibility in its structure when it binds an inhibitor or virus S-protein. Therefore, the conformational change could be triggered by altered amino acids as well. Although some of missense variants we analyzed are not directly located on the PPI surface, the altered amino acids could affect the binding of virus S-protein allosterically. Since the binding of the inhibitor inside the active site triggers ACE2 to enter a closed state from an open state through a huge conformational change, the altered amino acids of active site residue could cause a structural change of ACE2 more easily. Thus, the His378Arg amino acid change may not only reduce ACE2 peptidase activity, but also change the structure of a PPI area to affect S-protein binding. When S19 mutates to the helix “killer” proline, it may destabilize the most important helix to contact with S-protein. For SARS, the 24QAK to 24KAE mutant of ACE2 slightly inhibits interaction with spike glycoprotein [15]. Gly211Arg, Asp206Gly, Arg219Cys/His, Lys341Arg, and Ile468Val may affect the interactions across secondary structures. Therefore, their mutations may destabilize the local structure significantly. Ser547Cys may only affect the stability of one secondary structure, which may have a minor effect on the S-protein binding. As listed in Table 1, Procko [16] studied the virus binding abilities of 2340 human ACE2 mutants by using deep mutagenesis experiments. It shows that the Ser19Pro mutant is a strong booster for viral binding, and His378Arg is a weak booster. Thus, the local structural change from the Ser19Pro or His378Arg mutation may enhance the S-protein interaction allosterically based on his experimental results. It should be noted that our predicted deleterious variants in protein structure lack of experimental validation. Further exploration would be required to further confirm their potential effects on ACE2 function. It should also be addressed that, for theoretical predictions, different researchers provided different conclusions on the mutation effects based on different criteria and methods. For example, for the Ser19Pro mutation, it is predicted to be an interaction-booster by some groups [17–19], and an interaction-inhibitor by other groups [20, 21].

A recent genome-wide association study (GWAS) including 835 patients with COVID-19 and severe disease (defined as respiratory failure) and 1255 control participants from Italy, plus 775 patients and 950 control participants from Spain was conducted by the Severe COVID-19 GWAS Group [22]. They identified multiple genetic variants and genes associated with COVID-19 with respiratory failure. Although our study has characterized the putative functional and structurally related variants in ACE2 with top allele frequencies in various populations, the lack of phenotypes of COVID-19 prevents us from identifying susceptibility variants associated with a phenotype of COVID-19. However, our findings, together with other ACE2 genetic studies [17, 19], can prioritize the promising variants in ACE2 for further fast-track genotyping in blood samples from COVID-19 patients, which could provide a great opportunity to identify susceptibility variants in ACE2 related to symptoms of COVID-19 patients. On the other hand, our findings may also provide possible consideration of individuals carrying the identified variants in ACE2 for current vaccine development, especially those involved in ACE2 interaction with the S-protein of SARS-CoV-2.

## Conclusions

In this study, we characterized a total of 12 putative deleterious missense variants in the gene ACE2. Of those, we further provided strong evidence of nine missense variants that may disrupt the flexible regions of ACE2 protein structure or its protein–protein interaction with the RBD of S-protein of SARS-CoV-2. Results from this study highlight an important role of deleterious missense variants in the gene ACE2 that are present in the specific populations, which may affect SARS-CoV-2 recognition and infection. These variants could be important for the development of appropriate strategies of COVID-19 prevention, control and treatment to distinguish individuals between carrying and non-carrying those deleterious variants. Our findings may also provide a clue to partially explain why there were substantial discrepancies about the morbidity and mortality in regional disparity and distinct populations.

## Supplementary information

**Additional file 1.** A total of 251 non-silent genetic variants in ACE2 genes were characterized after removing those with low quality control, or had a AF = 0.

**Additional file 2: Figure S1.** Video of ACE2 open state vs close state. **Figure S2.** Video of SARS S-protein binds both ACE2 open and closed state.

## Data Availability

All characterized non-silent variants located in ACE2 are listed in Additional file 1 in our study. The possible impacts of an amino acid substitution on the structure/function from five protein prediction algorithms, including Polyphen-2 HumDiv, Poplyphen HumVar, Sorting Intolerant From Tolerant (SIFT), logistic regression test scores and MutationTaster, were conducted through ANNOVAR (https://doc-openbio.readthedocs.io/projects/annovar/). Our LiAn tool, developed in-house, will be available online in the future.

## Abbreviations

COVID-19: Coronavirus disease
SARS-CoV-2: Severe acute respiratory syndrome coronavirus 2
S-protein: Spike glycoprotein
ACE2: Angiotensin-converting enzyme 2
gnomAD: Genome Aggregation Database
LiAn: Legion Interfaces Analysis
PD: Peptidase domain
PDB: Protein data bank
PPI: Protein–protein interface
AF: Allele frequency
