# Supplementary material for: Investigation of the genetic variation in ACE2 on the structural recognition by the novel coronavirus (SARS-CoV-2)
Source: J Transl Med. 2020 Aug 24;18:321. doi: 10.1186/s12967-020-02486-7 (PMC7443814; doi:10.1186/s12967-020-02486-7)
Supplement: Supplementary file 2 — Additional file 2: Figure S1. Video of ACE2 open state vs close state. Figure S2. Video of SARS S-protein binds both ACE2 open and closed state. [file 12967_2020_2486_MOESM2_ESM.pptx]

## Slide 1
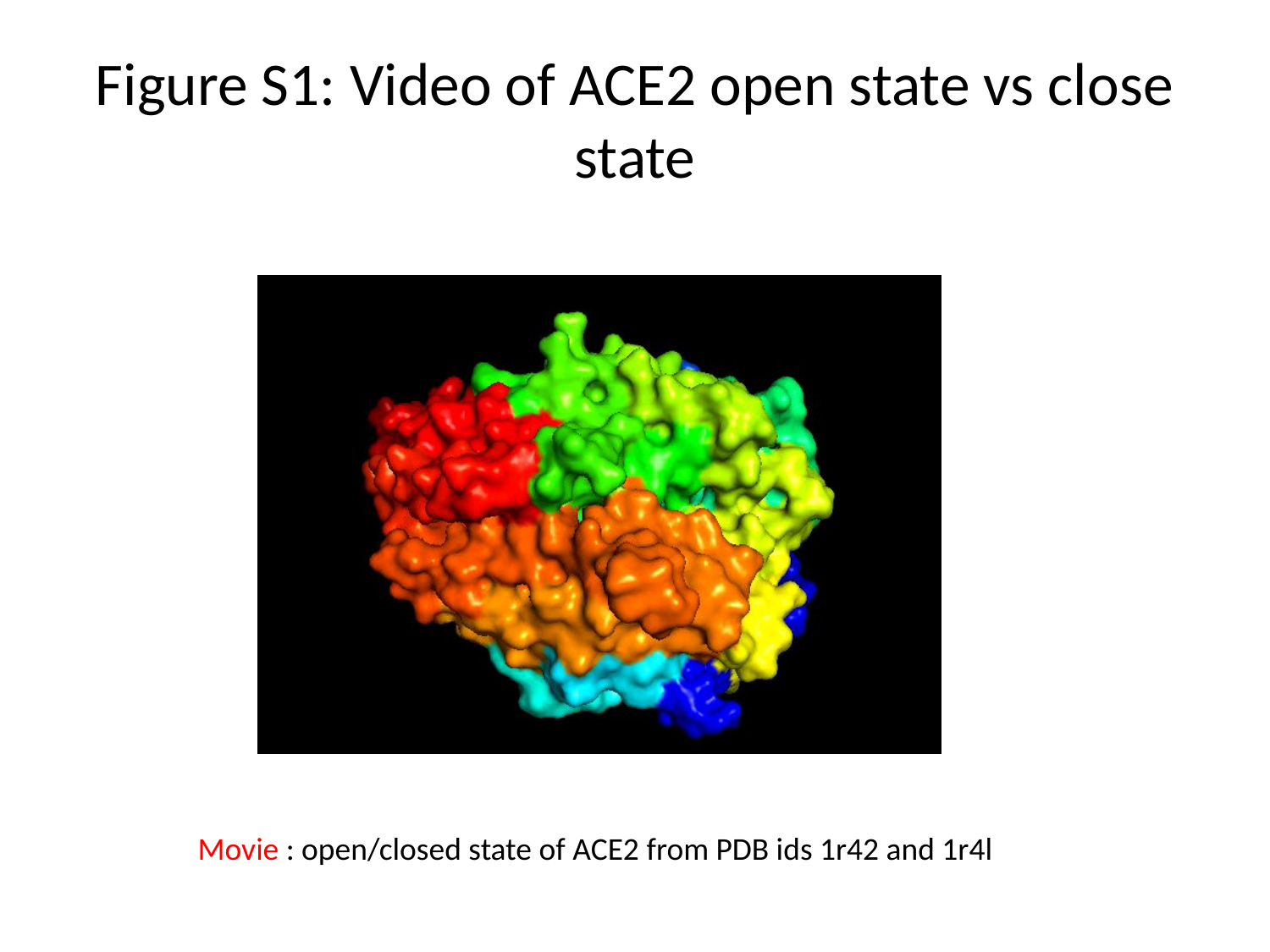

# Figure S1: Video of ACE2 open state vs close state
Movie : open/closed state of ACE2 from PDB ids 1r42 and 1r4l

## Slide 2
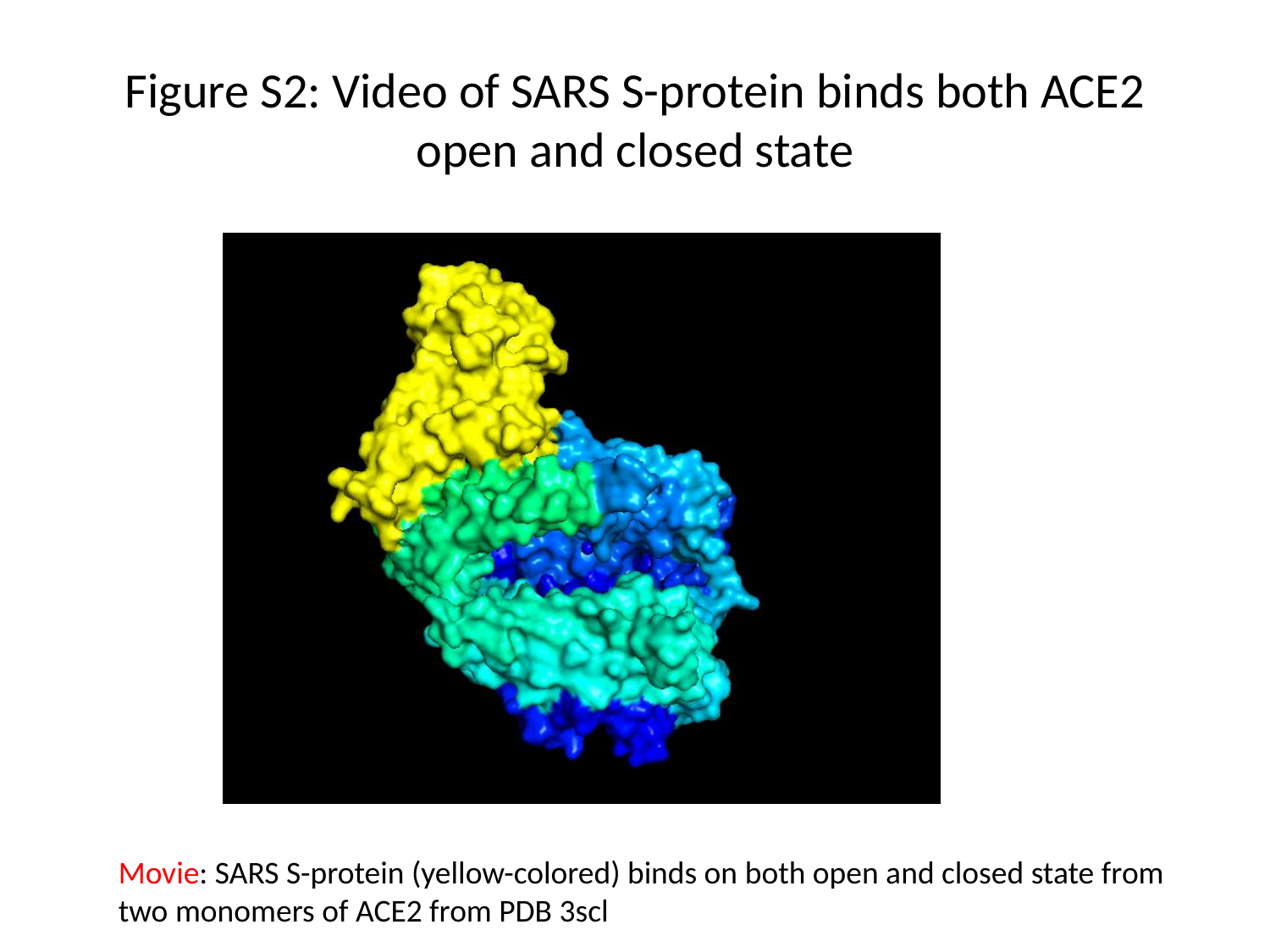

# Figure S2: Video of SARS S-protein binds both ACE2 open and closed state
Movie: SARS S-protein (yellow-colored) binds on both open and closed state from two monomers of ACE2 from PDB 3scl
